# Music-based interventions at workplaces: a scoping review

**DOI:** 10.1186/s12906-025-05221-1

**Published:** 2025-12-30

**Authors:** Israel Paul Nyarubeli, Bente E. Moen, Viggo Krüger, Brynjulf Stige

**Affiliations:** 1 Department of Psychosocial Science, University of Bergen, Bergen, Norway; 2 Department of Global Public Health and Primary Care, Centre for International Health, University of Bergen, Bergen, Norway; 3 GAMUT– The Grieg Academy Music Therapy Research Centre, University of Bergen, Bergen, Norway

**Keywords:** Music-based intervention, Stress, Well-being, Performance, Workplace, Review

## Abstract

**Background:**

Work-related health challenges such as work stress-related disorders, like anxiety and depression, are prevalent occupational health issues that lead to disability and an increased risk of chronic diseases. Various workplace interventions are used to reduce these health problems, but their effectiveness remains uncertain. Recognized for their health benefits, music-based interventions have the potential to reduce stress, improve physiological parameters and enhance workplace health and well-being. However, the effects of these kinds of interventions in preventing work-related health problems have not been extensively studied.

**Objective:**

This scoping review aimed to examine “What does the research literature tell about music-based interventions in the workplace?” Specifically, we synthesized the following: a) “What kinds of music-based interventions are used to reduce work-related stress and improve workplace performance and well-being?” and “Do studies indicate effects to reduce work-related stress and improve workplace performance and well-being?”.

**Results:**

This review revealed 25 studies showing potential effects of music-based interventions on well-being, stress reduction and productivity across various workplaces and 5 studies showing no effects. Music-based interventions, ranging from mindful music sessions to recreational music-making, consistently improved mood, reduced anxiety, and decreased stress levels. However, most study interventions have not been described in detail and are difficult to categorize. Objective outcome measures such as heart rate, blood pressure, and salivary amylase levels, indicate physiological benefits among workers. These findings suggest the potential and feasibility of music-based interventions in workplace settings.

**Conclusion:**

Music-based interventions have been implemented to address occupational stress, enhancing both psychological and physiological health benefits. However, the limited research in occupational settings necessitates further studies to optimize the design and feasibility of music-based interventions tailored to workplace needs.

**Supplementary Information:**

The online version contains supplementary material available at 10.1186/s12906-025-05221-1.

## Introduction

Good health and well-being are key aspects of enhanced productivity, satisfaction, and mental health in workplaces. Work-related stress disorders are among the major occupational health challenges reported globally, contributing to a large burden of pain, disability, and chronic disorders among working populations [40, 46]. The world of work is changing, causing stressful situations for employers as well as employees who are trying to adapt [22]. The World Health Organization estimated that 15% of individuals of working age had mental disorders in 2019 and that depression and anxiety resulted in a loss of about 12 billion working days per year [65, 66]. Recent trends indicate that stress levels are worsening among working population worldwide, with about 85% of countries reporting increased psychological stress between 2008 and 2021 [43]. Nine out of 10 workers reported to have chronic stress at work in the United States in 2022 [59]. However, the situation is likely to be devastating in many countries in the global south which lacks the necessary infrastructure, funding and expertise to conduct large scale and effective interventions on occupational health challenges including work related stress disorders. Furthermore, studies in this area are scarce, limiting evidence-based policy development and effective interventions.

Several interventions for the prevention and treatment of stress disorders have been designed and implemented, including for example, physical exercise, medication, and different therapies, but we still have limited knowledge on how these disorders can be reduced across the globe, and there are uncertainties regarding the effectiveness and feasibility of several of these programs [13, 23].

Music can be expressed as more than just a sequence of sounds; it is an active cultural design process that has evolved throughout human history (with both vocal and instrumental production of sounds – rhythm, melody, timbre, and harmony). It serves as a fundamental human expression within a semiotic system of sounds, involving the interpretation of sounds, silence, noises, and clamor, depending on the cultural context [64]. In this respect, we argue that workplace culture can accommodate music holistically [68]. It is believed that music evokes profound emotions, is often associated with significant life events, serves as a medium for expressing and processing emotions, facilitates altered states of consciousness, and provides a sense of meaning and connection [64]. Moreover, music, understood as artistic expression, aesthetic stimulus, and meaningful social and cultural activity, has been highlighted by the WHO in a scoping review on arts and health [17]. The rationale for examining the health resources provided by music and other art forms is grounded in several premises related to both policy and practice. At the policy level, the WHO highlights important developments in the global health policy arena, such as the health in all policies approach and the importance of multisectoral collaboration. At the practice level, the WHO highlights how music and other arts operate on several relevant levels at the same time, such as the individual, social, physical, and mental levels [17]. It could be argued that music and other arts-based interventions have unique potential to address the gamut of existing challenges that are recognized by researchers in relation to occupational stress. Music activities can function as multimodal health-promoting interventions with positive effects on health and well-being, especially when active participation in music is involved [61]. Music involves aesthetic engagement, imagination, cognitive stimulation, sensory and motor activation, evocation of emotions, social interaction, and a sense of community. They act in a complex mechanism that influences physical and mental health such as through psychological (emotional regulation, social bonding), neurobiological (dopamine-opioid reward systems), autonomic (stress reduction) and immune (anti-inflammatory pathways) [9, 15, 48]. These mechanisms suggest that music-based interventions could be relevant for workplace health promotion.

The literature on music-based interventions for stress reduction and improvement of well-being in the workplace remains limited and heterogeneous. While some music therapy interventions and other music-based interventions report positive effects on work stress reduction and well-being, their design, theoretical frameworks, or outcome measures for most studies differ. For example, a systematic review indicated that music-based interventions had a positive therapeutic effect on pain reduction among musicians [27]. In addition, a tailored 10-week exercise intervention conducted among musicians in Australia was found to be effective in reducing the frequency and severity of performance-related disorders [12]. On the other hand, music-based interventions have the potential to reduce stress and improve well-being by lowering heart rate and cortisol levels, decreasing heart rate and blood pressure (physiological responses, and releasing endorphins, (psychological responses,and enhancing participation (social responses [27]. Music-based interventions, including activities such as drumming circles and group singing, are widely used in clinical settings to promote emotional release and community bonding [18]. Music therapy interventions, facilitated by a professional music therapist, include receptive approaches (various forms of listening) as well as active approaches (singing, playing, improvising, composing) and are often also combined with various verbal approaches [35]. The interpretation of this heterogeneity is through the lens of complex music-based interventions in complex systems, where the intervention effect is contingent on context, mode of delivery, and mechanisms [54].

The literature referred to in the previous paragraph reveals that the study on the health affordance of music has developed into a highly interdisciplinary and diverse field with respect to the subfield of music-based interventions in the workplace. There is enormous variation in the choice of interventions, outcome measures, theories, and scientific rationale. This diversity is perhaps expected in such an interdisciplinary field. However, understanding similarities and differences in the approaches taken across studies is challenging because of substantial shortcomings in transparency and reporting quality in some studies [50]. In this work, we employ theory from the discipline of music therapy as tools for navigation in this complex conceptual landscape. For decades, music therapy theorists have problematized the tendency in some research studies to treat music as a stimulus or means only [1]. Human beings indeed react to music, but there are other levels of analysis to consider, such as the possibilities that music provides for human action and interaction [57]. Consequently, music therapy theorists have increasingly discussed music as an activity where context and culture, human interaction, and human agency matter [2, 24]. For some researchers focusing on the effects of interventions, this approach might sound overly theoretical, perhaps even confounding. To examine how such theorizing could be bridged with intervention studies, it becomes relevant, however, if one acknowledges that both interventions and the systems that they are part of might be complex [39].

Despite the background, the current review may also be anchored into synergistic theoretical grounds. First, music-based interventions or music therapy research suggest that receptive and active listening to music may affect regulation, attention, emotion arousal, social bonding, and work stress physiology (stimulus), with expert or therapy facilitated methods, i.e., guided imagery and music resulting in improved mood disturbances, reduced stress and well-being, and improved physiological parameters such as cortisol among workers as found in several studies [6, 13, 17]. Secondly, Job-Demand Resources (JD-R) model for occupational setting predict that autonomy and social support (resources) buffer the impact of job demands on strain (mood and stress regulation) and foster engagement and productivity (performance) [5]. The model provides for key characteristics of music-based interventions (active vs. receptive, solitary vs. collaborative) where more active and collaborative music activities may enhance positive outcomes, while receptive/solitary approaches may preliminarily target individual outcomes such as arousal and mood. Summing up, the two theoretical grounds complement each other where music – based interventions can be understood both as direct stimuli impacting workers’ physiology and emotions, and as resources within JD-R model that buffer stress and enhance engagement.

Therefore, the present scoping review aims to map and synthesize the existing literature on music- based interventions in the workplace settings, with particular attention to their reported outcomes such as reducing work stress, improving workplace performance and well-being, and their methodological characteristics [37]. Therefore, in this review, the research question is as follows: “*What does the research literature tell about music-based interventions at the workplace?”* We have examined two specific questions: *“What kind of music-based interventions are used to reduce work-related stress and improve workplace performance and well-being?”* and *“Do interventions potentially indicate reduction in work-related stress, improve workplace performance and well-being?”.*

## Methods

This study was designed as a scoping review to map evidence from the literature available on interventions aimed at reducing stress, improving performance, or improving well-being of employees in the workplace [37]. We covered the concepts of music therapy or music – based interventions. Relevant subject headings were selected for each database, and free text terms were used. The PRISMA extension for Scoping Reviews: Checklist and Explanation (PRISMA-ScR) [60], together with Arksey and O’Malley’s methodological framework for scoping review [3], were followed.

### Eligibility criteria and selection of studies

For the current review, primary/original studies were included if they met the following criteria under the Population, Intervention, Comparator and Outcome (PICO) framework and were published in English in a peer-reviewed journal. The target population included any individual or group (male/female), occupation, work, or employment. Music-based intervention (music therapy, music intervention, music genre or sound-medication or intervention). Comparator (no intervention, routine activity, work). Outcome (muscular skeletal pain-relief, stress reduction, workplace well-being, improved performance, productivity or quality sleep). The study design included randomized control trials (RCTs), non-randomized studies, cohort studies, longitudinal studies, cross-sectional studies, effectiveness studies, and interventional studies. There was no restriction on qualitative or quantitative studies. The search string used in each electronic database was (music therapy/or acoustic stimulation/) OR (music*.ti, ab, kf.) OR (auditory or audio or acoustic) adj3 (intervention* or therap* or stimul* or relaxation).ti, ab, kf.) AND ((work/or exp workplace/or employment/) OR ((work or working or workplace or occupational or professional) adj3 (climate or condition* or environment)).ti, ab, kf.) OR (employee*.ti, ab, kf)).

**Fig. 1:**
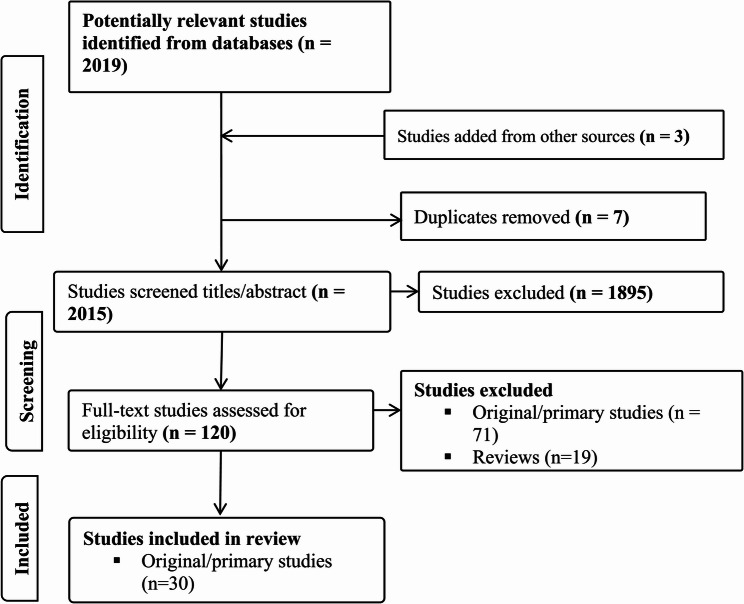
PRISMA -Flowchart: The inclusion and exclusion of publications of a review study on workplace music interventions conducted to reduce work-related stress and improve workplace performance and well-being

We conducted a search for eligible studies that were published from the early nineteenth century to 19th June 2024. The search was performed in the RILM abstracts of music literature, Ovid MEDLINE, OVID Embase, the Cochrane Library and Web of Science. The search of the electronic databases revealed 2019 potentially eligible studies. Three studies were added through backward citation searches of reference lists. Seven duplicates were removed using a fully automated AI-based deduplication solution, Deduklick [8]. A total of 2015 studies were exported into Covidence for the screening process (*Review Summary | Music interventions at the workplace | Covidence*). Two independent authors screened the titles and abstracts of the articles on the basis of the inclusion criteria. Both authors independently assessed the full texts of potentially eligible studies. Any disagreements were resolved by consensus. For title and abstract screening, 1895 studies were excluded, and 120 studies remained. Full-text screening excluded 71 original/primary studies and 19 review studies. The 71 studies excluded were animal studies/irrelevant study designs (*n* = 29), incorrect patient populations (*n* = 12), not published in the English language (*n* = 3), studies that did not report relevant study outcomes (*n* = 7), unrelated music interventions (*n* = 18) and those that had irrelevant settings (*n* = 2). Finally, 30 original/primary studies were included in this review, as shown in Fig. 1. Relevant studies were charted using web imbedded form, categorizing key information such as descriptions of basic study characteristics (author, year of publication, country, population/occupation studied), music-based intervention types and designs, outcomes measures (objective physiological measures, subjective, cognitive and performance and economic measures of outcomes), with reported methods of music—based interventions delivery. Key results were deductively synthesized narratively to highlight potential interventions relevant for prevention of occupational stress, improved productivity and well-being and other key reported outcomes categorized based on occupational and music theoretical foundations. Additionally, practical patterns and theoretical linkages that inform how music- based interventions can be applied in workplace health promotion were highlighted.

## Results

### Characteristics of the studies

This review included a total of 30 primary studies. Most of these studies were from Europe (13) or the Americas (10), whereas four studies were from Asian countries, two from African countries and one from Australia. The studies were published between 1967 and 2024. Most of the studies (16) were conducted in a wide range of working populations, such as various health care workers, factory workers, university staff and students, tailors, traffic controllers, bank employees, skateboard factory workers, call centers, and information technology (IT). Not all the studies described their working groups in detail. Furthermore, the review spanned a wide range of potential music-based interventions, with or without control groups, and the outcome assessment consisted of subjective (involving self-reported assessment tools such as stress, burnout, mood and anxiety), as well as objective tests such as improved memory capacity and attention to physiological parameters (blood pressure, heart rate, salivary amylase, cytokines, interleukin-10, natural killer cells and cortisol) (Table 1).

**Table 1:** Main characteristics of the included studies with different types of music-based interventions and reported outcomes (*N* = 30)

(First author, year) Country	Population	Intervention	Control group (Yes/No)	Outcome	Assessment of outcome O = Objective; S = Subjective
(Alqatari et al., 2022), Ireland	a) 54 university staff b) 9 participants	a) 30–45 min Mindful Music with live music sessions at lunchtime during work hours six times between March and December 2020 b) 4 prerecorded online sessions of 13 min between September 2020 and January 2021	No	Self-reported feelings and self-registered heart beats Unclear results. Maybe more well-being was obtained Live music was preferred	S
(Axelsen et al., 2022) [4], Denmark	623 healthy volunteers from different SME enterprises. 3 groups: 167, 152 and 140 (77 dropped out)	30 days—intervention of health volunteers a) Mindfulness b) Music listening c) Controls	Yes	a) Improvement in sustained attention, memory capacity and self-perceived stress b) Reduction in self-perceived stress	O and S
(Beck et al., 2015) [6], Denmark	20 Workers on sick leave 1) 13 2) 7	1) Guided imagery and music: 6 two-hour sessions 2) Waiting list for music sessions	Yes	Improved well-being, reduced stress, reduced anxiety, reduced depression, cortisol reduction	O
(Bittman et al., 2003) [7], USA	112 employees from a care retirement community center, divided into 2 groups First intervention, then no intervention	One hour of recreational music-making (RMM). One-hour recreational music-making for 6 weeks	No	Reductions in self-reported burnout and mood dimensions (6-sessions) Cost-effectiveness evaluation	S + Potentially projected cost saving 100-bed facility
Bravo-Herrera & Carazo, 2019) [10], Costa Rica	26 women and 27 men from IT company	Rythm therapy on mood	No	People become more mindful if they play music	S
(Calamassi et al., 2022) [11], Italy	54 health care providers in medical emergency unit station 3 groups: −1) 18; 2) 19; 3) 17	Listening to music for 10 min work break 1) 440 Hz; 2) 432 Hz;3) no music	No	Anxiety decreased in all 3 groups Group 2 respiratory rate and systolic blood pressure were reduced	O
(Delerue & Rabusseau, 2020) [14], Taiwan	20 oncology staff	A music listening program (chosen oneself) once a day at workplace, 20 min for 15 weeks	No	Improved well-being	S
(Duchemin et al., 2015) [16], USA	32 personnel in surgical care: 1) 16; 2) 16	1-h weekly mindfulness, yoga and music relaxation	Yes	Salivary amylase reduction No self-reported stress reduction	O
**(** Froschauer et al., 2023) [19]**,** Austria	1) 15 inexperienced medical students; 2) 15 experienced microsurgeons	Listen to music during surgery	Yes	No effect on time to completion or surgical result. Those who listened to music liked it	S
(Gatti & da Silva, 2007) [20], Brazil	49 staff at emergency medical service	Listened to music for the first 6 h of day shift for 2 weeks	No	41% experienced the music positively 15% experienced the music negatively	S
(Hohneck et al., 2024) [25], Germany	100 health care staff (healthy volunteers) 1) 50 2) 50	12 min of sound intervention once with classical music 1) with conventional headphones 2) with modified headphones	Yes	Reduced stress and reduced systolic heart pressure and increased well-being was seen for both types of headphones	O
(Huang & Shih, 2011) [26], Taiwan	89 voluntary workers at a university. 4 groups: 1) 23; 2) 22; 3) 20; 4) 24	Background music 1) Quiet (control); 2) Pop music 3) Classical music; 4) Chinese music	Yes	No difference between groups Improved attention among those who liked the music	S
(Jacobsen et al., 2022) [28], Denmark	699 citizens (329 Vaccinated on days with music and 370 on days without music), and 39 vaccination staff (23 on days with music and 16 on days without) Performed during covid-19 vaccination	Calming background music 3 days, and 3 days without music	No	Music was positive for mood for all and positive for collaboration with colleagues for staff	S
(Kacem et al., 2020) [29], Tunisia	Operating room staff	3 music listening sessions per day one month	No	Decrease of self-reported stress and burnout	S
(Klatt et al., 2017) [30], Denmark	27 employees in a large bank received intervention, 30 controls were on waiting list	8 weeks, 1 h per week with mindfulness, music and yoga combined	Yes	The mindfulness group experienced a reduction in self-reported stress level, better sleep and higher work capacity	S
(Klatt et al., 2015) [31], Denmark	34 staff in intensive care unit Controls were on waiting list	8 weeks, 1 h per week with mindfulness, music and yoga combined	Yes	Breath counts were reduced after sessions, resilience, work engagement and vigor were increased	S
(Ladenberger-Leo, 1982) [32], Poland	40 women with monotonous work	2 h of music (or not) in each shift for 1 month, 7 months observed	No	Higher productivity in months with music. Feeling better	O
(Yıldırım & Çiriş Yıldız, 2022) [67], Turkey	104 nurses a) 52 b) 52 controls	a) Mindfulness-based breathing and music listening 30 min b) Controls relaxed for 30 min – 1 session only	Yes	Reduction in stress level and work-related strain. Increased psychological well-being	S
(Lesiuk, 2008) [34], USA	33 air traffic controllers An unknown number of people in the Control group	a) Preferred music listening b) Controls were sitting in silence	Yes	Self-reported stress levels were reduced. Blood pressure and heart rate have not changed	S
(Lesiuk, 2005) [33], Canada	56 computer information system developers from 4 IT companies	Listening to music after own choice when the workers wanted 4 weeks; 2 with music, 1 without music, 1 with music	No	Positive effect on work quality and time-on-task	O
(Newman et al., 1966) [38], USA	26 Skateboard factory workers at assembly lines	2 h daily with music of 4 types versus days with no music, 5 weeks	No	No change in productivity, but the workers liked to listen to music	S
(Onyara & K'Obonyo, 2017) [41], Kenya	3 garment factories: 119 tailors from each factory	1) Background music all day 2) Background music was put on and off 3) No music	Yes	Positive effect on the performance of the tailors The tailors loved listening to music	O
(Ploukou & Panagopoulou, 2018) [44], Greece	48 oncology nurses 1) 22- intervention 2) 26 -control	1-h music class per week; playing music themselves 4 weeks	Yes	Reduction in anxiety, depression and psychosomatic symptoms	S + Unclear number in the control group
(Popkin et al., 2011), [45], USA	10 or 20 nursing staff in cancer wards. Only 8 answered a survey	45 min therapy with music in a ‘remembrance ceremony’ for patients	No	Music increased effectiveness in the grief process	S
(Rubbi et al., 2024), [51], Italy	122 nursing staff from surgical team from 3 hospitals 1) Music; 2) No music	Italian music 80% of the time, 20% other music types during surgical procedures	Yes	Anxiety and stress were slightly reduced among music exposed workers	S
(Shih et al., 2016), [53], Taiwan	31 males 34 females On-job students at a university 1) 33; 2) 32	a) Attention tested without background music b) Tested with background music 1) Music with lyrics; 2) Music without lyrics	Yes	Attention was higher when listening to music without lyrics Background music with lyrics negatively impacted the attention	S + Unclear how intervention took place and how often
(Smith & Erkkila, 2008), [55], Australia	80 call center workers 1) 40 2) 40	1) A single 15 min guitar music relaxation session 2) Controls participated in a discussion about stressful calls	Yes	Reduced anxiety	S
(Steinberg et al., 2016), [56], USA	32 Surgical intensive care workers Participant numbers were unclear	Yoga with music 1 h per week, 8 weeks	Yes	Increased work satisfaction, interpreted as a stress reduction	S
(Wachi et al., 2007), [62], Japan	40 factory workers 1) 20 2) 20	1) One-hour recreational music making session 2) One-hour leisure reading. Crossover after 6 months	Yes	Enhanced mood, lower stress-induced cytokine interleukin-10 and higher natural killer cell (NK) activity	O
(Wlodarczyk, 2013), [63], USA	68 hospice workers 1) 34 2) 34	1) A single 1-h session of music-based intervention for grief resolution (active music-making and songwriting)	Yes	Music group improved on a scale on personal sacrifice burden No difference in burnout or fatigue	S

Among the 30 studies included in the review, 25 showed generally positive effects on the measured or reported outcomes. Nine studies did not have a control group. In addition, a clearly described randomization of the participants to the intervention or control group was observed in twelve of the studies. Two of the studies had a blinded randomization of participants into an intervention group and a control group, and most studies had low numbers of participants, ranging from 16–40 in the groups (Table 1).

Many of the studies included in this review were music-based interventions conducted among health personnel. For this group, the interventions were most often passive listening to music during a break, during a short period of the day, or as background music during surgical work. Additionally, several of these studies included an intervention consisting of music listening combined with other activities, such as yoga and mindfulness (Table 1).

Four studies included workers with monotonous working situations, such as working at an assembly line in a factory. Three of these studies indicate a relationship between music-based intervention and increased productivity. The music-based interventions were of different types.

Only one included study incorporated a form of clinical and potential economic analysis of music-based interventions. In a randomized, controlled study of 112 interdisciplinary long-term care workers, evaluated a six session recreational music-making (RMM) program (group drumming with keyboard accompaniment) and, using an independent consultant’s economic model, projected annual savings of $89,100 for a typical 100-bed facility, with an extrapolated industry-wide estimate of $1.46 billion in annual savings if implemented broadly [7]. These projections were linked primarily to reductions in staff turnover and were reported alongside statistically significant reductions in burnout and total mood disturbance (TMD). Nevertheless, the analysis was model-based and was not derived from prospectively observed costs within the trial. In addition, the study model did not report standard health economic evaluation parameters (e.g., incremental cost-effectiveness ratios, costing methods, or sensitivity analyses). Thus, while suggestive of potential cost implications, the available data were deemed insufficient to support economic conclusions beyond hypothesis-generation.

### Music-based interventions

The reviewed literature shows a large diversity of music-based interventions conducted in various occupational contexts. Descriptions of the interventions often lack details, making them difficult to categorize. Additionally, among various studies, the same term might reflect different concepts, or various terms might reflect similar concepts. A comprehensive and accurate categorization of the interventions in the reviewed literature is therefore not possible. Informed by the music therapy theory we referred to in the introduction, we highlight two dimensions that seem to reflect basic differences in the reasoning that informed the design of music-based interventions. First, in many studies, music was treated as a stimulus, whereas in *some* studies, music was employed as an activity. Second, in many studies, interventions were designed so that employees were affected or involved in solitude. Even though the setting could be a work environment with many people, the music-based intervention did not involve much human interaction. In other studies, however, human collaboration was a central characteristic of the intervention.

**Fig. 2:**
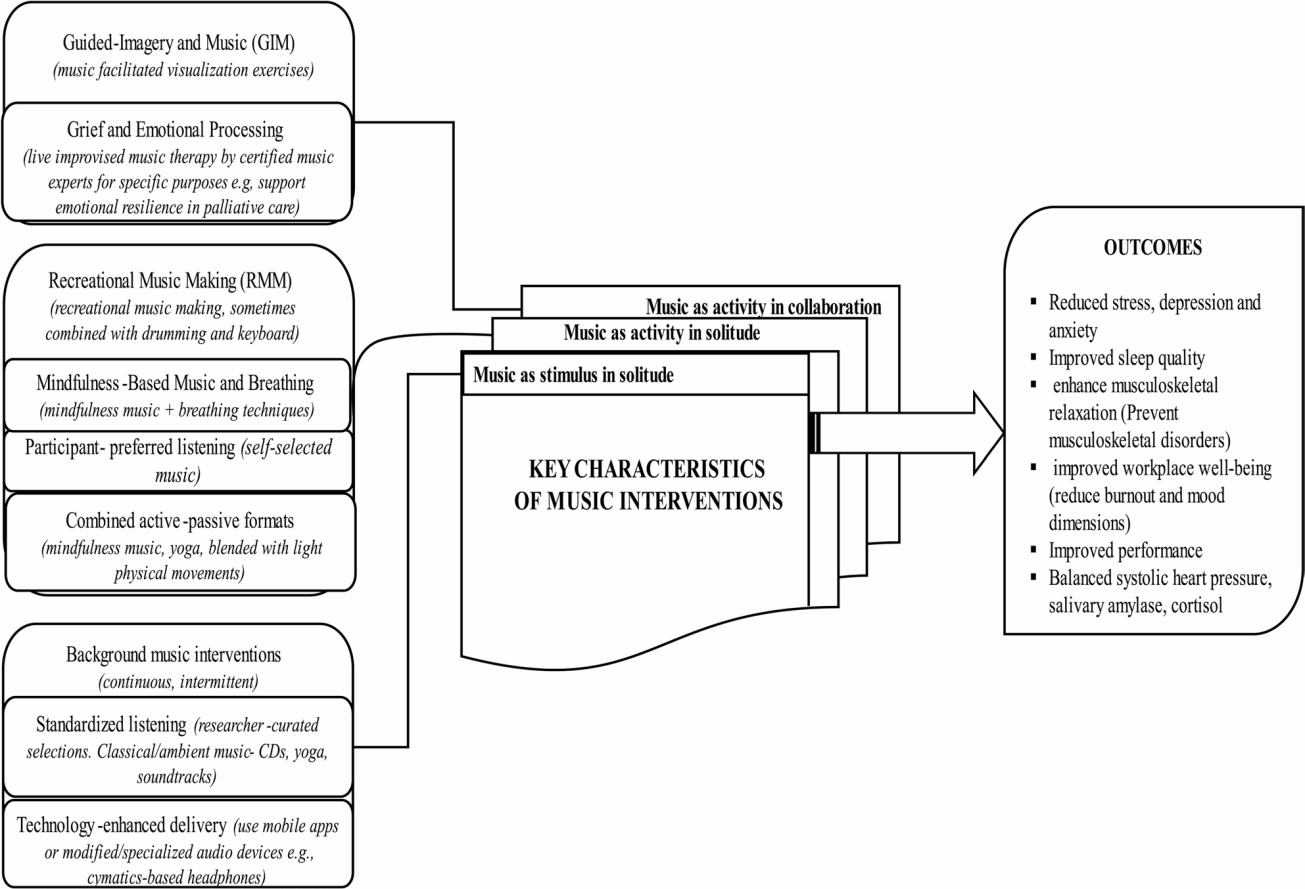
Summary of key characteristic of music-based interventions, subcategories and outcomes in the Scoping review

Given that human collaboration without activity is hard to imagine, this provides us with three categories of intervention: a. Music as a stimulus in solitude. b. Music as an activity in solitude. c. Music as an activity in collaboration. These categories do, of course, not represent the reviewed literature in a perfect or accurate way, since there are nuances and combinations of various sorts, but they highlight key characteristics that allow for comparison and analysis. Figure 2 presents these key characteristics in the context of the diversity of interventions and outcomes in the reviewed literature. We will exemplify and describe this briefly.

#### Music as a stimulus in solitude

Passive music listening interventions exemplify this first category and are aimed primarily at enhancing mood, reducing stress, or influencing cognitive performance. Music listening was the most frequently studied intervention type in the literature, typically involving sessions of individuals listening to music with no active participation. The subcategories included background music interventions, standardized listening, and technology-facilitated delivery. Studies have shown that music listening can positively influence attention and emotional well-being, though the effects on outcomes vary. For instance, Huang (2011) conducted a study with 89 voluntary workers in Taiwan, where participants exposed to background music demonstrated increased attention and positive work engagement, provided that they found the music enjoyable. Conversely, Shih (2016) reported that background music with lyrics negatively impacts attention performance among university students engaged in occupational tasks, indicating that the type of music plays a critical role in shaping work outcomes.

#### Music as activity in solitude

Music as an activity might involve listening if the employee is allowed to choose music on the basis of personal preference. For example, in the study of Lesiuk [33], the participants could choose the music they liked from a library provided by the researcher, which included genres such as contemporary pop, oldies, country, jazz, new age, and classical pieces. Active music engagement also involves direct participation in music-making activities, such as singing, drumming, or instrument playing, which are typically facilitated in a group setting. There were relatively few such studies in our sample, but these interventions seemed to have an effect. For instance, Plokou (2017) examined the effects of structured music classes for oncology nurses in Greece and reported that four one-hour music sessions per week significantly reduced anxiety, depression, and psychosomatic symptoms. The music classes were performed in groups, so there might also be effects of human interaction (see next category), but this is not highlighted in the researchers’ description of the intervention. Similarly, the interventions studied by Yıldırım’s and Yıldız in Turkey (2022) were performed in groups, but only each individual’s attention to breathing, body sensations, thoughts, and emotions were focused on when the intervention was described, not the interaction and collaboration among participants. This mindfulness-based music listening intervention was performed among nurses and demonstrated reductions in work-related strain and increased psychological well-being.

#### Music as an activity in collaboration

Practitioner-facilitated interventions, often facilitated by trained music therapists, usually employ music as an activity in collaboration. They also represent a more formal approach to integrating music into occupational health. There are only a few studies in this category in the present study, and they focus on the therapeutic and health-promoting potential of *listening* to recorded or live music in a practitioner-facilitated session. Interventions that facilitate collaborative music-making are not well represented in the reviewed literature. The music therapy interventions represented in the reviewed literature employ tailored listening activities, including Guided Imagery and Music (GIM), with the aim of addressing specific health outcomes, such as stress reduction, mood regulation, and physical well-being. Beck et al. [6] conducted a randomized controlled trial on the effects of GIM on work-related stress and reported significant improvements in mood disturbance, physical distress, and cortisol levels. Popkin et al. [45] designed a music therapy-centered grief intervention for nurses and ancillary staff working in cancer settings. Two music therapists provided music on harp and guitars, improvised and lived, which enabled the music therapists to adjust to the situation and follow cues from the participants. Researchers have reported the positive impact of music therapy on grief processing among cancer ward care workers, underscoring the role of music therapy in supporting the emotional well-being of healthcare professionals.

### Outcomes after intervention

Overall, the reviewed studies reported varying outcomes regarding the potential effects of music-based interventions. Several studies included self-reports related to mental health issues such as stress, burnout, mood and anxiety, and they indicated potential improvements in these self-reported parameters (Table 1). Five of the studies did not show any effect of the intervention on the outcome, and these five studies are not described further.

#### Objective outcomes

Nine studies used objective tests of different types as outcomes. Three of these studies indicated that attention and/or memory capacity were improved by intervention. Different types of work productivity were measured in four of the studies, and the intervention improved productivity in three of the studies. Six studies measured one or more objective physiological parameters: blood pressure, heart rate, salivary amylase, cytokines, interleucine-10, natural killer cells and cortisol. Five of these studies concluded that the intervention had a positive effect on physiological parameters among workers (Table 2).

**Table 2:** Studies with objectively measured outcomes

(Author, year) Country	Population	Intervention	Outcome of music-based intervention
(Axelsen et al., 2022) [4] Denmark	SME	30 days—intervention of health volunteers a) Mindfulness b) Music listening c) Controls	Improvement in sustained attention and memory
(Beck et al., 2015) [6] Denmark	Different workers	1) Guided imagery and music: 6 two-hour sessions 2) Waiting list for music sessions	Reduced cortisol
(Calamassi et al., 2022) [11] Italy	Health care workers	Listening to music for 10 min work break 1) 440 Hz; 2) 432 Hz;3) no music	Reduced respiratory rate and systolic blood pressure
(Duchemin et al., 2015) [16] USA	Healthcare workers	1-h weekly mindfulness, yoga and music relaxation	Reduced salivary amylase
(Hohneck et al., 2024) [25] Germany	Health care workers	12 min of sound intervention once with classical music 1) with conventional headphones 2) with modified headphones	Reduced systolic heart pressure
(Ladenberger-Leo, 1982) [32] Poland	Monotonous work	2 h of music (or not) in each shift for 1 month, 7 months observed	Improved productivity in months with
(Lesiuk, 2005) [33] Canada	IT companies	Listening to music after own choice when the workers wanted 4 weeks; 2 with music, 1 without music, 1 with music	Improved work quality and time-on-task
(Onyara & K'Obonyo, 2017) [41] Kenya	Tailors	1) Background music all day 2) Background music was put on and off 3) No music	Improved performance
(Wachi et al., 2007) [62] Japan	Factory workers	1) One-hour recreational music making session 2) One-hour leisure reading. Crossover after 6 months	Lower stress-induced cytokine interleukin-10 and higher natural killer cell (NK) activity

## Discussion

The review found promising positive effects of various music-based interventions on psychological outcomes, such as mood, reduced anxiety, decreased stress levels and improved performance and productivity, as well as on physiological processes, such as heart rate, blood pressure, and salivary amylase, among various working populations. Despite this, the effectiveness of these interventions varies widely, partly due to differences in design, implementation, and evaluation methods for outcomes (subjective vs. objective) and categorization of music-based interventions. Nevertheless, the findings suggest that music-based interventions hold potentials as low-cost, non-invasive, and culturally versatile tools for promoting health and well-being in workplaces. That these types of interventions may lead to beneficial effects is supported by a study by Viola et al. [61], who wrote a review of the role of music in promoting health and well-being in general. However, this study was not focused on interventions at workplaces.

There are very few previous literature reviews similar to our study, which focused on workplace interventions. However, our findings are in line with those of a previous literature review from Italy [47], which also showed that there are few original studies in the area. This study describes 14 studies and concludes that psychological factors, communication, rehabilitative outcomes and cognitive and work performance were the main outcome variables. Unfortunately, the music-based interventions were frequently sparse, and other broader search terms than in our present study were used. Similarly, a synthesis of two multilevel meta-analyses conducted by de Witte et al. [13] suggested that music-based interventions had an overall effect on both physiological and psychological stress outcomes. The authors, however, noted theoretical and methodological challenges related to operationalization of the concept of stress and categorization of music-related variables, inconsistence of variables across studies, stressing the need for harmonizations of protocols for music-based interventions for generalized results. Furthermore, a review by Marine et al. [36], which was updated in 2023, concluded that limited evidence is available for workplace interventions to reduce occupational stress among health care workers, highlighting methodological weaknesses and variability in study design analogous to the current review [36, 58].

Descriptions of the music-based interventions in our present study often lacked details, and terms and concepts were used inconsistently. This has also been reported in several other reviews described above [13, 47, 58]. If not addressed, this will represent a significant barrier to advances in research on music-based interventions, and it will also impede implementation and practice development. In addition, this mirrors longstanding concerns about intervention reporting in music- based research and underscores the value of standardized, theory informed descriptions of context, participant roles, methods of delivery and intended outcomes. Robb et al. [49] addressed similar concerns many years ago and provided guidelines for researchers designing and describing music-based interventions (these guidelines have recently been revised, see [50]. We do see the relevance of providing guidelines as one way of addressing these limitations in the research literature. This might remind researchers that they need not only to state that they have used a music-based intervention but also to outline it carefully, with descriptions of context and culture,participants, roles, and relationships; activities and artifacts; goals and rationale; and so on. Given the inherent interdisciplinary nature of workplace music interventions, greater integration of theory and music may be necessary to ensure the design and implementation of music-based interventions align with their intended mechanisms of action.

After having examined the literature, our appraisal is, however, that there is a need for something more than and different from refined guidelines and increased guideline adherence. Studying music-based interventions in workplace settings is inevitably an interdisciplinary enterprise. With some notable exceptions, the studies we reviewed demonstrate that there is potential for much improvement in advanced and high-quality interdisciplinarity in this field. Many studies seem to be performed with little awareness of advances in theory development within music therapy and related fields. Consequently, the music’s potential as a collaborative, interdisciplinary activity is underrepresented in the literature that we reviewed. From a complex-system perspective, workplace music interventions interact with local contexts (task or job demand, autonomy), delivery mode (facilitation, music selection), and mechanism (emotion regulation, social support). Variability in these parameters plausibly explains variabilities in outcomes across studies and settings [54].

The reviewed literature underscores the multifaceted role of music in occupational settings, with evidence suggesting potential benefits for employee well-being, work satisfaction, and stress reduction. While passive music listening may facilitate mixed effects on productivity, it may consistently contribute to mood enhancement and stress alleviation. Active music engagement may foster social connection, creative expression, and psychological decompression, offering valuable support in high-stress occupations. Practitioner-facilitated interventions demonstrate potential in addressing work-related stress and facilitating mental health recovery, albeit requiring broader implementation and evaluation.

Given that few studies have used active music engagement as an intervention, we cannot draw clear conclusions. However, the studies might be important, as examples of what type of music-based intervention has given a positive result. Also, it must be remembered that music-making initiatives have other interesting effects on the workplace as well, and may offer opportunities for creative expression, team-building, and psychological decompression, ultimately fostering a more resilient and cohesive workforce [42].

The quality of the selected studies in this review was not systematically evaluated. This could have been done systematically, but as the studies were so diverse, guided by scope of the current work, i.e., *Scoping Review*, we decided to show diversity, despite methodological flaws. However, we noted that there are studies with the label *‘randomized controlled trials.’* The randomization in these studies may have occurred among the individuals employed at a work site. However, randomization of workplaces was not performed and was not even discussed in the studies. Some studies were performed with blinding, but far from all. Additionally, several studies were performed without control groups, and the results cannot be relied upon. Lastly, several studies had very small sample sizes. In total, the findings suggest the potential and feasibility of music-based interventions in workplace settings. This suggests integrating music-based programs into employee health promotion and wellness initiatives to enhance mental health and reduce burnout, with a focus on personalized approaches to maximize benefits.

The variability in findings across different intervention types highlights the importance of considering contextual factors, such as job demands, task complexity, and employee preferences, when music-based approaches are integrated into occupational health strategies.

In the current review, robust economic evaluation of workplace music-based interventions is largely absent. The only identified analysis was single study and was modelled (turnover-based projections) with no reporting of standard elements such as cost categories, incremental cost-effectiveness ratios, or sensitivity analyses rather than a full trial based economic evaluation [7]. We suggest that caution should be taken when interpreting these results. Future research should focus on optimizing intervention designs, exploring long-term effects, and examining the economic implications of music-based interventions in workplace settings. The findings related to passive music listening as an intervention suggest that the effects of background music on workplace productivity are contingent upon personal preferences, task complexity, and the type of music being played. Music listening has also been associated with reducing workplace stress and fostering a positive work environment. Sanseverino et al. [52], found that the use of music in occupational settings was positively correlated with job satisfaction and perceived productivity. This is particularly relevant in high-stress occupations, where background music may serve as a subtle yet effective stress-relief mechanism. However, despite the positive implications for employee well-being, the relationship between this type of passive music listening and direct productivity outcomes remains inconclusive, necessitating further research in diverse workplace contexts.

Additionally, in this scoping review, none of the included studies explicitly measured or reported the influence of background industrial noise on music-based intervention delivery or outcomes. This represents a significant gap, particularly for factory and high-noise environments where sound exposure from machinery, alarms, and ventilation systems may affect both the audibility of music and its psychological impact. From an occupational health perspective, noise is a recognized stress-related hazard and a contributor to fatigue and cognitive load [21, 65, 66]. Introducing music-based interventions without accounting for baseline noise levels could lead to masking safety signals, hearing strain, or reduced intervention fidelity. Based on this gap, future research should take this aspect into consideration.

In conclusion, the current scoping review shows that music-based interventions have been implemented in addressing occupational stress disorders, enhancing psychological resilience, and promoting faster recovery in high-demand work environments together with improvements in physiological parameters. However, the limited research in occupational settings necessitates further exploration to optimize the design and feasibility of music-based interventions tailored to workplace needs. In addition, future research should explore the dynamic relationships among music, employers, and the broader work environment, incorporating both small-scale participant-centered qualitative investigations and larger-scale quantitative analyses to capture a greater picture of the impact of music in workplace contexts. Such studies can support workplaces in promoting the well-being and health of employees.

## Supplementary Information

Supplementary Material 1.

## Data Availability

The information analyzed during the current study is available from the corresponding author upon reasonable request.

## Abbreviations

GIM: Guided Imagery and Music
PICO: Population, Intervention, Comparator and Outcome
RCT: Randomized Controlled Trial
RMM: Recreational Music Making
WHO: World Health Organization
